# Freeware tool for analysing numbers and sizes of cell colonies

**DOI:** 10.1007/s00411-018-00772-z

**Published:** 2019-01-23

**Authors:** Beata Brzozowska, Maciej Gałecki, Adrianna Tartas, Józef Ginter, Urszula Kaźmierczak, Lovisa Lundholm

**Affiliations:** 1 Department of Molecular Biosciences, Centre for Radiation Protection Research, The Wenner-Gren Institute, Stockholm University, Stockholm, Sweden; 2 Biomedical Physics Division, Institute of Experimental Physics, Faculty of Physics, University of Warsaw, 5 Pasteura Street, 106 91 Warsaw, Poland; 3 Heavy Ion Laboratory, University of Warsaw, Warsaw, Poland

**Keywords:** Clonogenic cell survival assay, Colony counter, Colony size histogram, countPHICS

## Abstract

The clonogenic cell survival assay is a basic method to study the cytotoxic effect of radiation and chemical toxins. In large experimental setups, counting of colonies by eye is tiresome and prone to bias. Moreover, it is often interesting to quantify the size of individual colonies. Such analyses are largely facilitated by computerised image analysis systems. Although a number of such systems exist, they all focus on enumerating colonies and not on analysing the colony size. We have developed a new software package for both counting colonies and plotting their size distributions. The software called count and Plot HIstograms of Colony Size (countPHICS) consists of two parts: (1) a macro written for ImageJ which analyses computerised images of cell culture dishes or 6-well plates, counts colonies, estimates their size and saves the results in a text file; (2) a program written with QT Creator which reads the text file, plots histograms of colony size distribution and fits the best function. The full program is freely available at: http://www.fuw.edu.pl/~bbrzozow/FizMed/countPHICS.html. In conclusion, our new publically available software will facilitate colony counting and provide additional information on the colony growth rate, which is relevant especially for radiosensitisation studies.

## Introduction

The clonogenic cell survival assay (CCSA) is a basic method used in radiation biology and toxicology to estimate the cytotoxic effect of physical or chemical toxins. It was introduced in 1955 by Puck et al. (Puck et al. 1956) following the discovery that cells grown under in vitro conditions require conditioned medium if they are to form colonies from a single precursor (Sanford et al. 1948). CCSA was instrumental in discovering such essential phenomena in radiation research as the dose rate effect (Hall and Bedford 1964), lethal and sublethal radiation damage (Elkind et al. 1967) and the variable radiosensitivity of cells in different phases of the cell cycle (Sinclair and Morton 1966). As compared to most assays which are used to assess the genotoxic or cytotoxic effects of various agents, the great advantage of CCSA is that it measures the ability of cells to retain their reproductive integrity after a prolonged period of time, usually between 1 and 2 weeks following exposure (Franken et al. 2006). Hence, cells have time to express phenotypic effects which require time and possibly several cell divisions for development. Already in 1964 Sinclair discovered that ionising radiation induces some heritable lesions which lead to colonies of small size (Sinclair 1964). This observation was confirmed and pursued by Beer (Beer 1979; Beer and Szumiel 1994). Later, it was suggested that the heritable lesions are a manifestation of genomic instability (Pampfer and Streffer 1989).

While the colony number can be quantified by eye, the analysis of colony size requires some kind of measurement. Traditionally, a dense conglomerate of cells is regarded as a colony when the number of cells exceeds 50 (Franken et al. 2006), which corresponds to seven cell divisions assuming no cell death. Counting of cells in a colony can be carried out with an inverted microscope but this is time consuming. The area of a colony can be estimated by measuring its diameter but this assumes a round shape which is often not given. Thus, there is a need for an image analysis tool which would estimate the areas of colonies, best in an automated and high throughput setup that is supplemented by a statistical analysis of the results.

A number of automated counting programs have been developed (Barber et al. 2001; Cai et al. 2011; Dahle et al. 2004; Geissmann 2013; Lamprecht et al. 2007) but they all focus on enumerating colonies and not on a systematic analysis of colony sizes. Such a tool was developed by us and is described herein. It consists of a macro written for ImageJ which analyses computerised images of cell culture dishes and a program which plots histograms of colony size distribution and fits the best function. The program countPHICS is freely available at: http://www.fuw.edu.pl/~bbrzozow/FizMed/countPHICS.html.

## Materials and methods

The software tested for colony size distribution analysis is divided into two parts: the macro which allows us to measure the size of the automatically (or semiautomatically) counted colonies and the program dedicated to draw distributions of colony sizes in the form of histograms. Analysing the distributions by their shape and parameters gives additional information to the clonogenic survival studies.

### Colony counter

By choosing the “Perform image processing” option from the countPHICS introductory window, the macro written for the ImageJ software (version 1.49v, Java 1.8.0_45, Wayen Rasband, U.S. National Institutes of Health, Bethesda, MD, USA; website: http://rsb.info.nih.gov/ij/download.html) will be applied. As a regular text file it can be modified with any available editor. It can also be opened with ImageJ and used directly from its menu. If the processed pictures are properly named (according to the instruction included in Electronic Supplementary Materials), the macro can be executed for them all simultaneously.

The available ImageJ macro files dedicated to the colony counting were already introduced and discussed elsewhere (Cai et al. 2011). However, the macro *counter.txt* within countPHICS includes additional features such as the Gaussian blurring parameter described below, as well as batch analysis where many images are processed at once and the size of each colony is saved to a text file. This file is then used for colony size analysis in the newly developed PHICS part of the program. There is no need to install ImageJ or additional plugins since the software works stand-alone and its interface is created to allow a step by step-instruction. The countPHICS gives a possibility to analyse the colony size when using the CCSA and this feature is introduced for the first time. The major functions used in the countPHICS macro will be discussed briefly in this section.

**Fig. 1:**
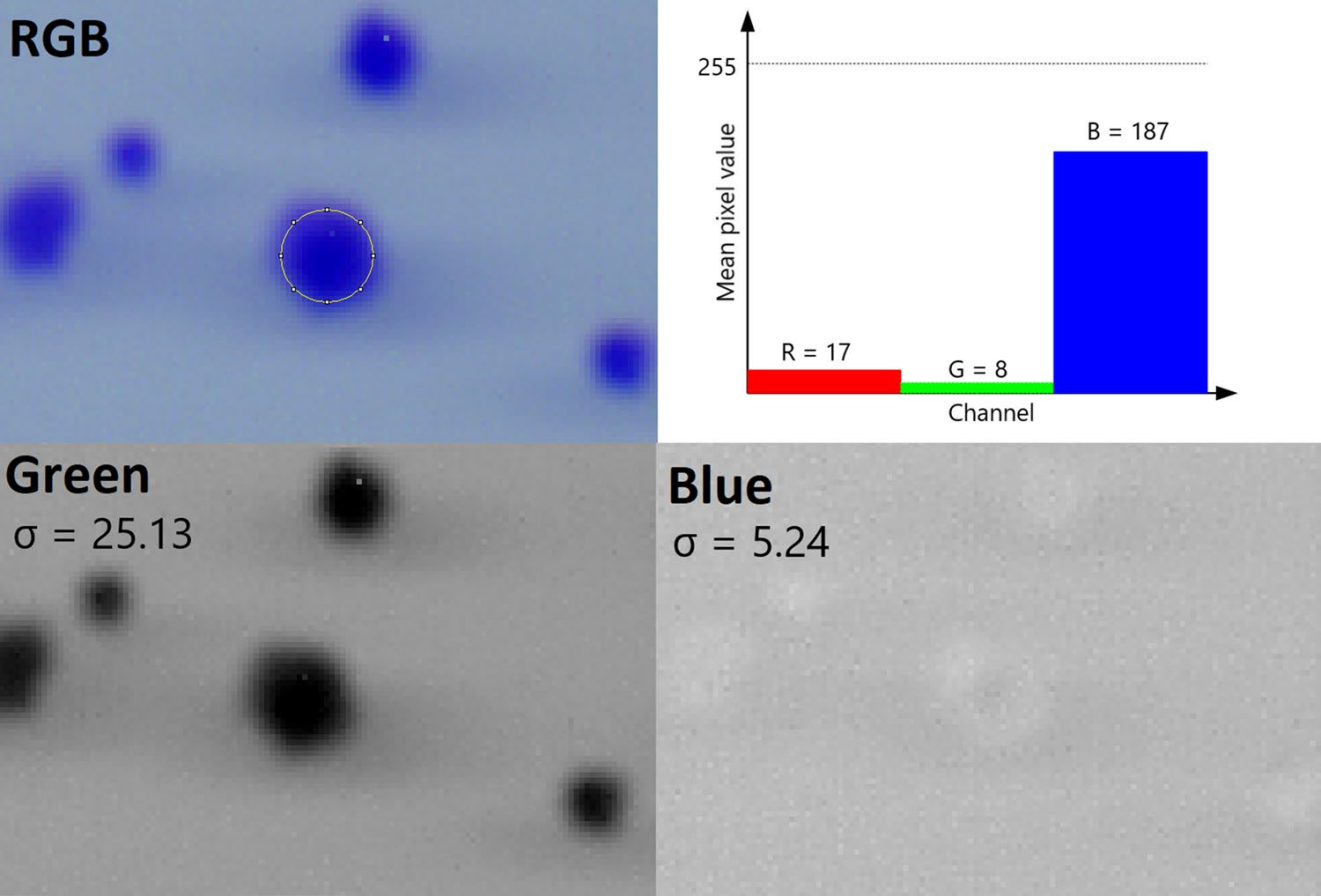
Comparison of the pixel value distribution for different stack image components. White colour in RGB scale is (255, 255, 255) while black is (0, 0, 0). The first channel is red (R), the second—green (G) and the third—blue (B; RGB). To obtain the highest contrast, the channel where the average colony pixel value differed most from the background was selected. As shown in the RGB image, the colony is mostly blue in this case, which is also displayed in the graph. In this particular image, background was more white than black (closer to (255, 255, 255) than (0, 0, 0)), therefore the channel with the lowest average pixel value was chosen. In our case, it was green. The lower figure panels demonstrate that the green channel indeed gives far better contrast than the blue. In countPHICS, the standard deviation (σ) parameter was chosen for channel selection, because it distinguishes which channel differs the most from the background in general. (Colour figure online)

To reduce the background noise and expose the colonies more, the scanned plate picture is split automatically into the red, green and blue channel (RGB stack). Afterwards the image with the highest standard deviation (i.e. contrast of the colonies relative to the background) is used for further analysis (see Fig. 1). As an example, in this experiment, the green channel was used, and the red and blue ones were not needed.

There are two parameters which should be estimated to start the analysis and therefore set automatically or manually by the user in the dialog windows. A randomly chosen plate including some colonies should be used for this purpose. The first parameter is *sigma* which is the standard deviation of the Gaussian blurring filter. It is chosen as default depending on the image resolution. If the *sigma* value is too high the colonies might become less visible and their size will drastically increase. If it is too low the background noise will not be reduced correctly and during the analysis process some artefacts might be classified as colonies. The purpose of setting the *sigma* value, which corresponds to the smoothing process, is to reduce noise and eliminate artefacts of the image, and to make the colony colour more homogeneous.

The second parameter—rolling ball radius is used to remove smooth continuous background noise from the whole plate using the rolling ball algorithm of background subtraction. This parameter should be at least as large as the largest diameter of the object (given in pixels) that is not part of the background. While setting the value of *rolling ball radius* too high does not affect the analysis much, setting it too low can occasionally make the colony less visible resulting in a smaller number of colonies. The smoothing operation is needed for the rolling ball algorithm to work efficiently.

**Fig. 2:**
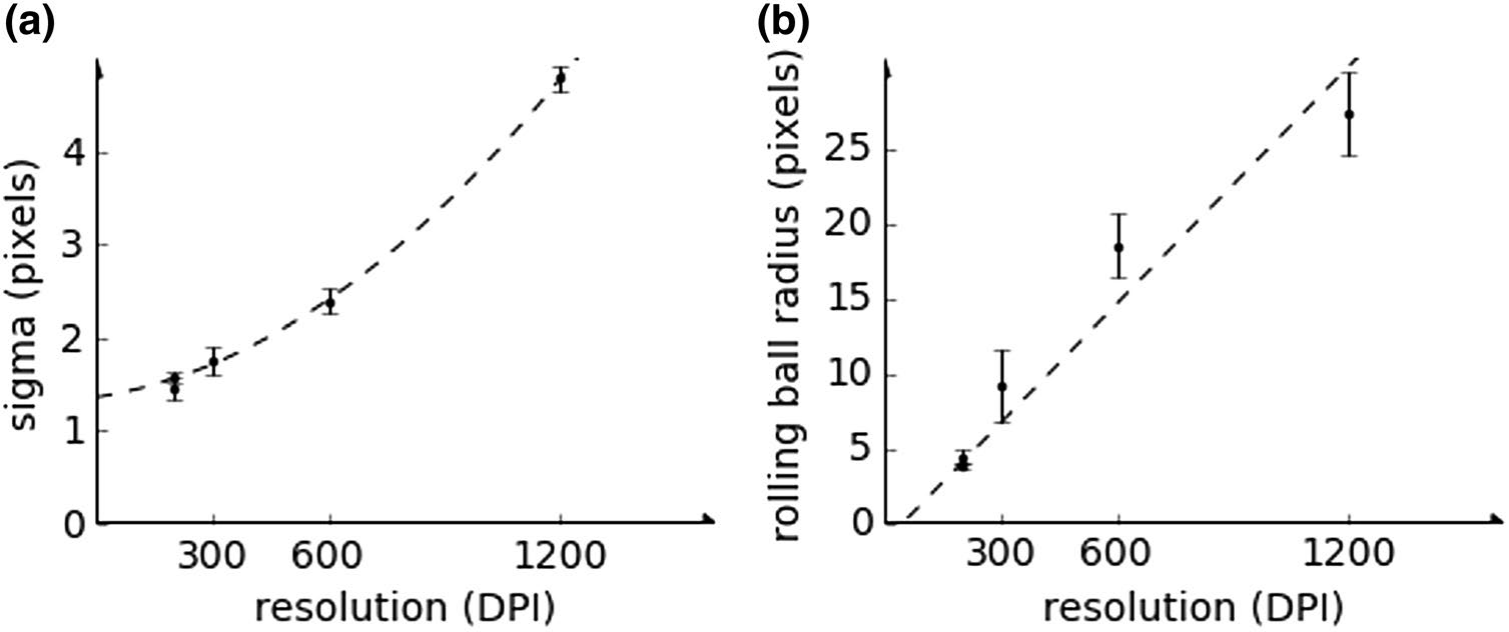
Parameter *sigma* (standard deviation of the Gaussian blurring) and *rolling ball radius* (background subtraction) were optimised to get the best agreement between number of colonies counted manually and automatically in relation to image resolution (dots per inch, DPI)

In the preparation of the automatic settings, both parameters were optimised to produce the best agreement with the colony numbers counted manually. The optimised parameter values are presented in Fig. 2 as a function of image resolution in terms of dots per inch (DPI). The relation between optimised *sigma* parameter and image resolution can be described by the polynomial function fitted according to the following formula:

$${\text{sigma}} \simeq 1.9 \cdot {10^{ - 6}} \cdot {x^2}+6.3 \cdot {10^{ - 4}} \cdot x+1.3,$$

where *x* is the image resolution. Optimal values of the *rolling ball radius* parameter tend to be approximately proportional to the image resolution (with a proportionality coefficient of 0.025).

Once the desired objects are maximally exposed, symmetrical and homogenous, the image can be converted to 8-bit format using an optimal *threshold* value. The value of this parameter can be also set automatically or manually. Pixels with a value above the threshold are set to black, while values below the threshold are set to white. The *threshold* value should be set in such a way that the converted black colonies are of similar size as the original ones. At the same time, black objects created from the background noise should be reduced as much as possible. If all the photos were taken under the same conditions, the average brightness of the images should be relatively equal assuming similar staining intensity. Therefore, the *threshold* value can be set at the start of analysis, and should be the same for each image.

Once the binary image is obtained the appropriate region of interest (ROI) should be fit, with the same size for every image. The ROI should be placed with a little margin away from the border of the dish, to make sure the edges of the dish will not be counted as a cell colony.

**Fig. 3:**
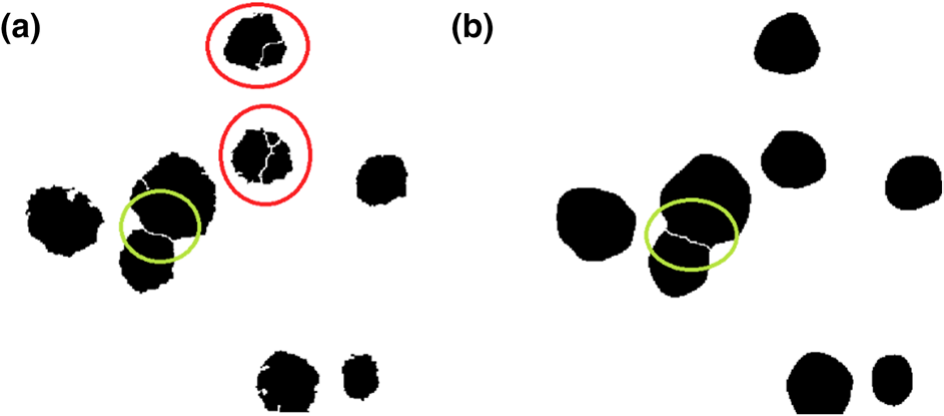
Watershed algorithm effect in the original picture (**a**) and the picture after Gaussian blurring (**b**). Green contours show the proper separation of the merged colonies which appear independently of Gaussian blurring application. Without Gaussian blurring, the watershed algorithm incorrectly divides a single colony into pieces (red contours). (Colour figure online)

Before the analysis process, one last step has to be done—the watershed segmentation. It is a process of splitting merged colonies as shown in Fig. 3 for original and pre-processed images.

Additionally, *minimal colony size* and *circularity* values are predefined. Only colonies with the area in the range specified by the size parameter will be detected. *Circularity* parameter is defined by the following formula: $4\pi \frac{{{\text{area}}}}{{{{{\text{(perimeter)}}}^{\text{2}}}}}$. By increasing the lower limit of the circularity range, colonies that are not round enough will be ignored. The last step of the macro is to measure areas of all the selected colonies and save them in the text file. The text files are an input for distribution analysis available with the software described in the next section.

When the parameters are chosen they should not be changed meanwhile unless the quality of pictures changed significantly.

### Plotting histograms of colony size

**Fig. 4:**
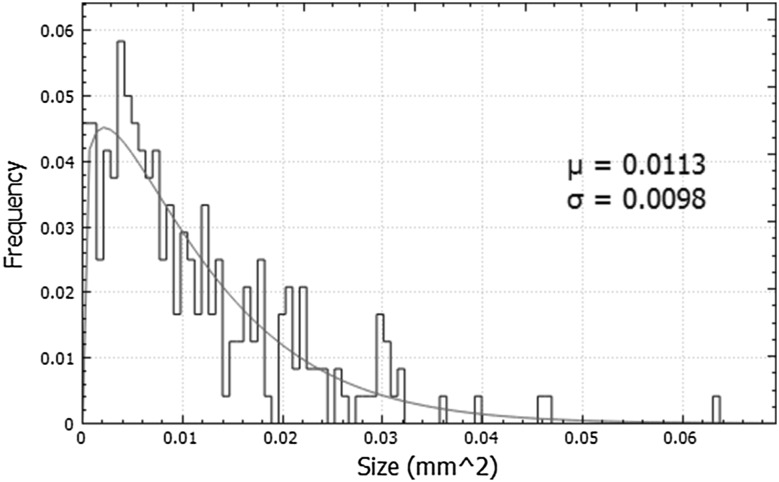
An example of a normalized colony size histogram with calculated Weibull parameters: mean value (μ) and square root of variation (σ)

To plot the histograms of colony size, our custom-made program was used. The software was written in C++ with the graphical user interface (GUI) created using the QT framework (Galassi et al. 2015) and its subroutine QCustomPlot 2016 (QCustomPlot online). For calculations, the program uses Gnu Scientific Library (GSL). GSL library was used to create a histogram and to fit the Gaussian and Weibull distribution to the data. The histogram contains a number of bins which count the events from a given range of a size variable (size intervals). The countPHICS uses multidimensional nonlinear least-squares fitting [Levenberg–Marquardt algorithm (Gill et al. 1981)] to estimate two parameters of the Gaussian and Weibull distributions (as shown in Fig. 4).

The formulae describing the Weibull distribution is given by

$$f\left( x \right)=c \cdot \frac{a}{{{b^a}}} \cdot {x^{a - 1}} \cdot {\text{exp}}\left( { - {{\left( {\frac{x}{b}} \right)}^a}} \right).$$

There are two parameters fitted using this function, *a* and *b*. They are needed to calculate the mean size value (µ) and the variation (*V* = σ^2^) (Mendenhall and Sincich 1995), shown in the countPHICS output picture.

The Gaussian distribution parameters are mean value (μ) and standard deviation (σ) according to the following equation:

$$f\left( x \right)=\frac{1}{{\sqrt {2\pi } \cdot \sigma }} \cdot {\text{exp}}\left( { - {{\left( {\frac{{x - \mu }}{{\sqrt 2 \sigma }}} \right)}^2}} \right).$$

The detailed countPHICS instruction (see Electronic Supplementary Materials) is uploaded together with the software itself on the webpage: http://www.fuw.edu.pl/~bbrzozow/FizMed/countPHICS.html.

### Cell culture and clonogenic survival assay

For program testing, three cell lines were used: human non-small cell lung cancer (NSCLC) cell lines H1299 and A549 (ATCC, Manassas, VA, USA) and Chinese hamster ovary cells (CHO-K1). H1299 and A549 cells were cultured in RPMI-1640 medium supplemented with 2 mM l-glutamine, 10% foetal bovine serum and 1% penicillin–streptomycin (Sigma-Aldrich, Stockholm, Sweden). Cells were plated at a density of 250 or 500 cells per 10 cm-plate and were incubated for 10, 12 or 14 days at 37 °C. CHO-K1 cells were cultured in McCoy’s 5A Medium (Gibco, USA), containing 10% foetal bovine serum (FBS) (Gibco, USA), 1% penicillin–streptomycin (Gibco, USA). CHO-K1 cells were irradiated with 0.1 Gy of ^12^C ions with an energy of 17 MeV and LET = 640 keV/µm. Non-irradiated CHO-K1 cells were plated at a density of 300 per 10 cm-plate, while irradiated cells were plated at a density of 600 per 10 cm-plate. CHO-K1 cells were incubated for 7 days in a humidified atmosphere at 37 °C with 5% CO_2_.

To provide an example of colony size analysis, a new analysis was performed on previously published data (Lundholm et al. 2014) where cells were pretreated or not with 10 µM mitogen-activated protein kinase kinase (MEK) inhibitor U0126. Inhibitor treatment was performed from 1 h before irradiation with 2 Gy and was kept during the first 72 h. A549 tumour initiating cells (TICs) were established from A549 cells by culture in sphere-forming conditions (non-adherently, in serum-free medium) and irradiated using a Co^60^ source as described previously (Lundholm et al. 2013, 2014).

After incubation, all samples were washed with phosphate-buffered saline (PBS). Cells were concomitantly fixed and stained using 5% Giemsa in 25% methanol for 10 min. Plates were washed using water and allowed to dry upside down, leaning towards the lid of the plate. Colonies containing over 50 cells were first manually counted, and then plates were filled with potato flour to improve the contrast and scanned according to Supplementary materials. The sizes of the colonies were measured manually with a ruler and automatically using countPHICS. The main aim of this study was to establish and verify the use of countPHICS, the use of different irradiation modalities and cell lines was due to their availability at different sites.

## Results

**Fig. 5:**
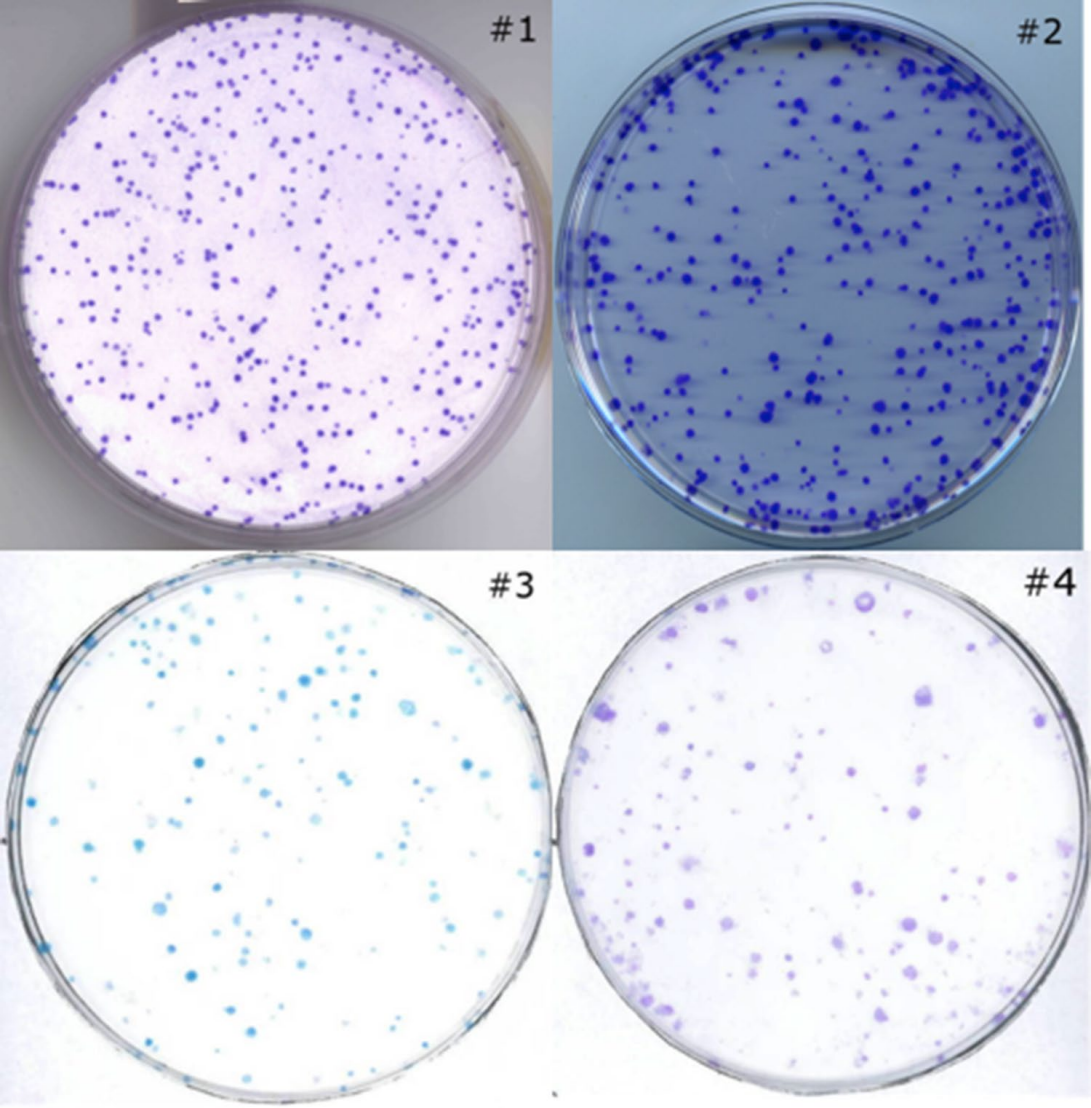
Representative images of the chosen categories: #1, #2, #3 and #4

The software described in this manuscript can be used to count colonies as well as to measure their size by plotting the size distributions and fitting the appropriate functions, thus allowing mean size values to be estimated. Four sample categories of differing quality were chosen for analysis based on possibility of distinguishing individual colonies and the colony contrast of the photo (Fig. 5). These categories were: an optimal distinguishability and a high contrast of colonies (#1), an intermediate distinguishability and a high contrast of colonies (#2), with an intermediate distinguishability and a low contrast of colonies (#3) and with a poor distinguishability and a low contrast of colonies (#4). Between 6 and 15 images were used for each category, where the number of colonies was measured (as shown in Table 1) and the size distributions were prepared using automatic and manual methods. The default settings (automatic *threshold* option, *sigma*, *rolling ball radius, minimum colony size* and *circularity*) were established for countPHICS to obtain good agreement between the number of colonies measured automatically and manually. In a typical analysis of CCSA data, the default settings are recommended to obtain optimal results.

**Table 1:** A comparison of relative differences in colony number counted between automatic (*N*_auto_) and manual (*N*_man_) scoring methods for different samples

Sample description	Number of analysed pictures/colonies	(*N*_man_ − *N*_auto_)/*N*_man_
#1: Optimal distinguishability and a high contrast of colonies	12/5651	0.023 ± 0.017
#2: Intermediate distinguishability and a high contrast of colonies	11/2447	0.070 ± 0.035
#3: Intermediate distinguishability and a low contrast of colonies	15/1571	0.25 ± 0.22
#4: Poor distinguishability and a low contrast of colonies	6/1316	0.123 ± 0.067

The diameter of a single colony was measured manually with a ruler in ImageJ program using the scanned images. Based on the assumption that each colony is a circle the size of a single colony in the plate was calculated according to the formula for circle area *π*⋅(diameter/2)^2^. The histograms of colony size created with manually measured values were compared with the size data analysed in the automatic way.

**Fig. 6:**
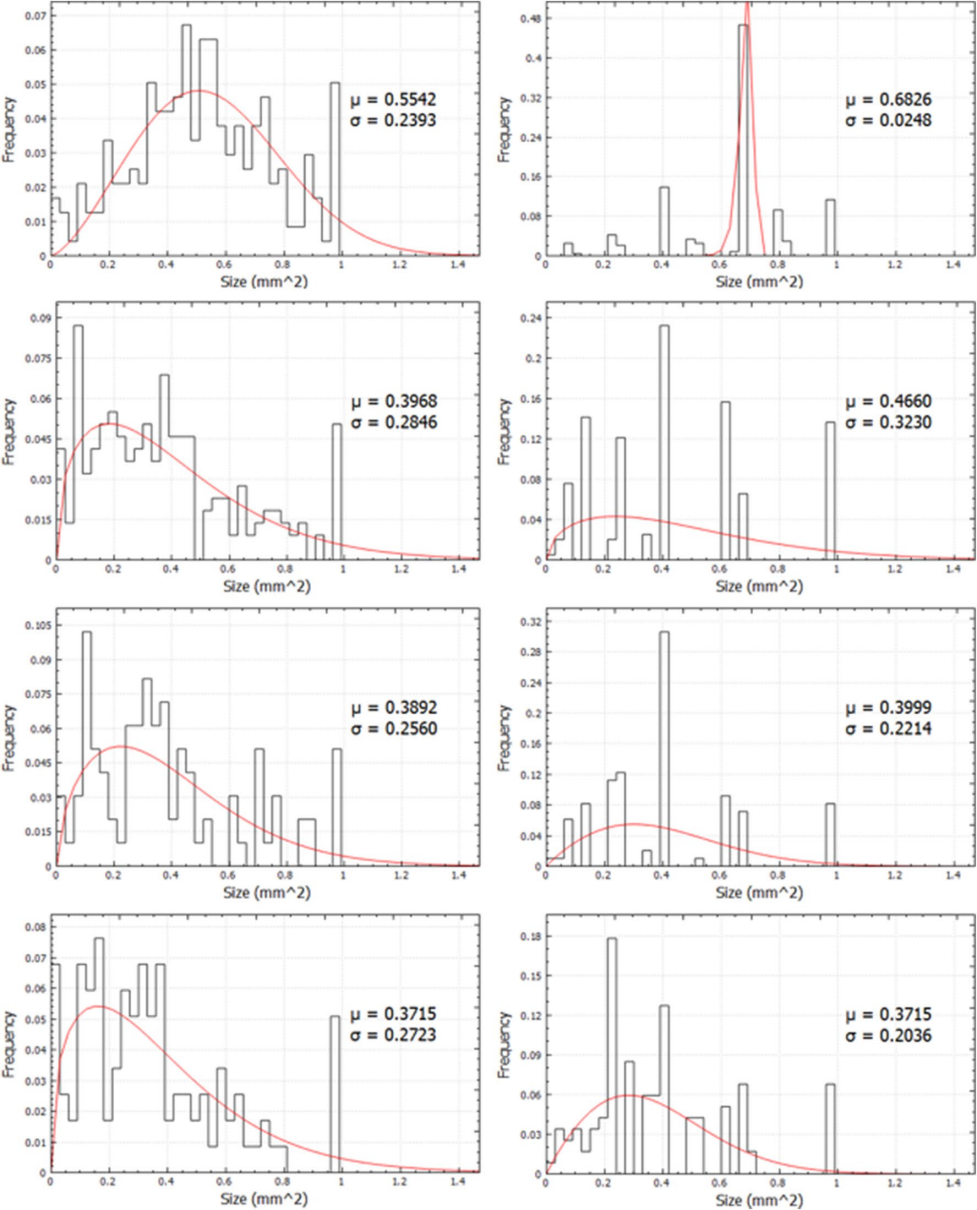
The size of colonies scored automatically (left) and manually (right) and compared with the Weibull fit for four different sample categories: #1, #2, #3 and #4. The number of analysed plates per sample category is given in Table 1

As shown in Fig. 6, for both methods the size values were not distributed normally, that is why the asymmetric Weibull function (and not Gaussian) was fitted. The Weibull and Gaussian distributions are described by the equation discussed above. To correctly draw the final conclusions, we decided to use the same fitted function type for all analysed data. Although there is a higher level of inaccuracy for the manual measurements and many assumptions had to be made, the comparison of fitted mean values looks reasonable. The shapes of histograms of the automatically counted colonies were smoother than for those counted manually, and appeared more reliable.

**Fig. 7:**
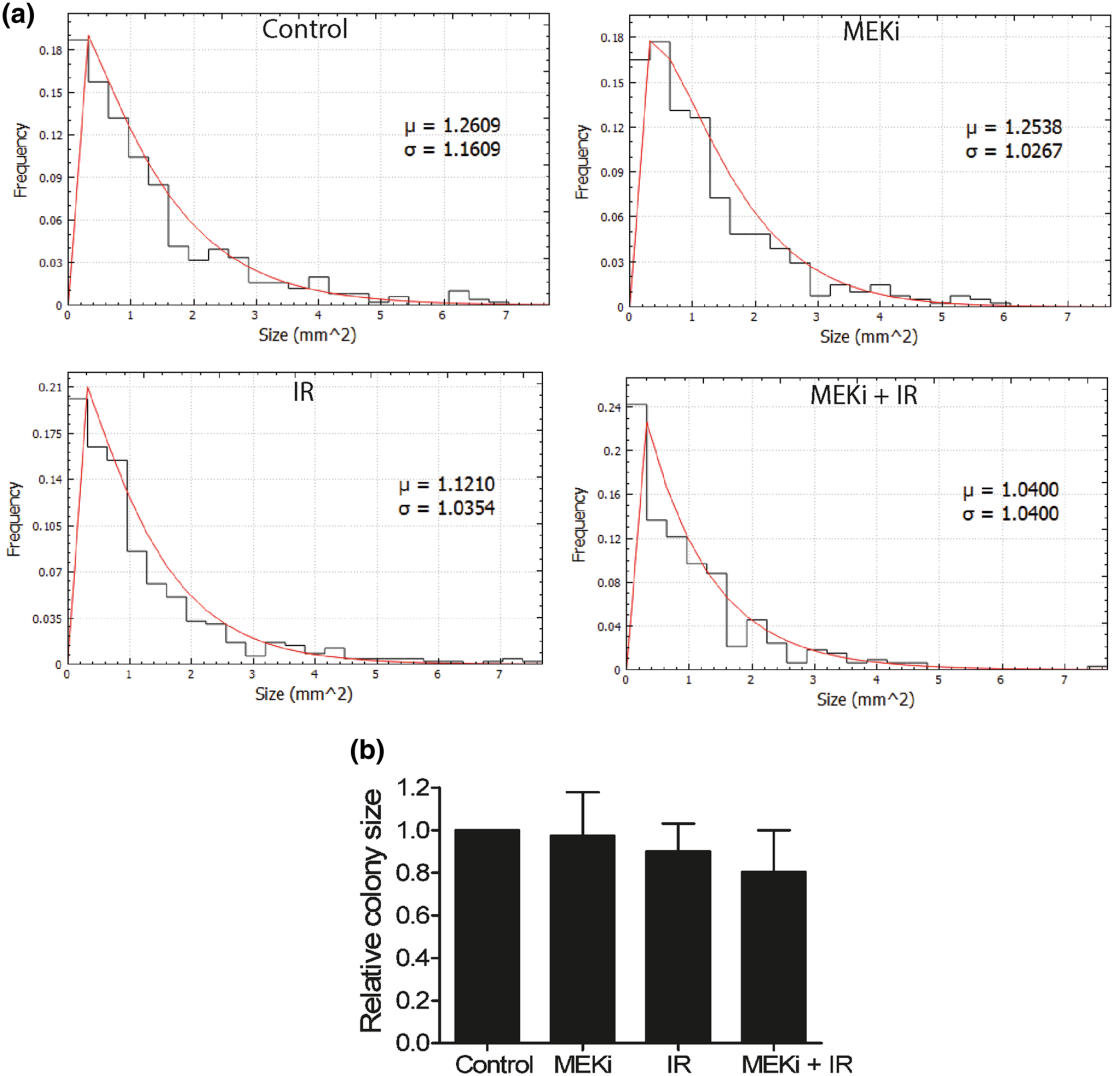
Histograms using colony sizes for all 3 experiments (**a**) and the mean colony size (+/-standard deviation, 3 experiments) (**b**) for A549 tumour initiating cells (TICs): untreated, after MEK inhibition, gamma irradiation (2 Gy) and the combination of these two. A reduction in growth rate is observed

A reduction in growth rate was observed but not quantified in mitogen-activated protein kinase kinase inhibition studies in non-small cell lung cancer TICs (Lundholm et al. 2014, Supplementary data). To provide an example of this additional feature of CCSA the countPHICS software was used to analyse the colony size using scanned plates where the colony number was already published (Lundholm et al. 2014). The colonies of A549 TICs after MEK inhibition, gamma irradiation or the combination were analysed and there was a trend towards a reduction in colony size for the cells pretreated with MEK inhibitor prior to irradiation (Fig. 7). Therefore, the colonies were not only reduced in number (Lundholm et al. 2014) but also at the level of growth rate (i.e. colony size) (Fig. 7).

In conclusion, the colony size analysis tool developed here helps to extract more information and provide an improved understanding of results from clonogenic assays, which is of potential importance for a large number of studies using radiation as well as other toxic compounds, in particular when using combinatory approaches.

## Discussion

Survival curves have been measured for many established cell lines grown in culture. Except for the biological variation between experiments, the analysis relies on the quality of the plates which are scored and used to prepare the plots. Since at least three independent experiments are required, commonly performed in duplicate, the number of colonies needed to be analysed is high which justifies the use of automatic support. The *counter.txt* as a part of the countPHICS software allows analysis of the data in a fast and unbiased way. Even if there are some colonies missing, especially when the edges of plates are impossible to be reliably scored and therefore neglected in the analysis, the same error propagates for all analysed samples. It protects the analysis from human impact, which is difficult to recognize and avoid. After parameter estimation (Gaussian blurring, background and threshold), many images can be processed at once and treated in exactly the same way. The comparison between the colony number counted manually and automatically show similar results, according to our test samples. We used 10-cm dishes, but in principle all types of round plates or dishes could be used for the analysis.

Further analysis of the colonies by measuring their size gives additional information, not incorporated in the clonogenic survival assay. The long lasting growth disturbances can be observed based on either the growth rate or the colony size. Quantitative analysis is possible with the countPHICS software presented here, allowing to plot the size distribution and fit Weibull or Gaussian functions including statistical calculations such as mean and standard deviation. Based on survival assay and using countPHICS software, descriptions on how the radiotherapy, chemotherapy or targeted therapy affect not only the number of colonies, but also their size, can be determined. The reduction in growth rate as an apparent additional feature of MEK inhibition in non-small cell lung cancer tumour initiating cells was already mentioned in (Lundholm et al. 2014), but there we did not have any tool to measure it quantitatively. Another example of reduced colony size was reported after combined MEK and EGFR/HER2 inhibition in breast cancer cells (Gayle et al. 2013). The quantitative description was possible to perform when using the software presented here. Our first report using the countPHICS software was for assaying the effect of histone deacetylase inhibitor (HDACi) pretreatment before irradiation of non-small cell lung cancer TICs (Eriksson et al. 2017), where HDACi pretreatment produced a reduction both in colony number and size.

Some of the improvements in our colony counting method compared to the previously published macro version from 2011 (Cai et al. 2011) is that the default parameter values are being optimised for images with different resolutions to get the best accuracy, also for pictures < 600 dpi. As described using the example pictures in the Instructions, the main limitation with this program is not the software per se, but the quality of the plates. The most troublesome examples are plates which are too crowded with colonies. Other less optimal cases are when plates had an insufficient incubation period (creating colonies that are difficult to detect) or too long an incubation period in which case the central part of the colony tends to fall off during fixation and staining. The latter example may be circumvented by modulating the circularity parameter.

## Conclusions

In conclusion, we present a new method to extract, visualise and statistically handle data using the combination of a macro for the well-established ImageJ program together with a newly written countPHICS program. In this publically available tool, a new application (colony size) is implemented, to be used for a variety of purposes in radiation, toxicology or cancer research.
